# Spatial Response Discrimination May Elicit a Simon Effect on a Non-Complementary Task

**DOI:** 10.1177/00315125231215854

**Published:** 2023-11-27

**Authors:** Melanie Y. Lam, Romeo Chua

**Affiliations:** 1 Department of Human Kinetics, 1270 St Francis Xavier University, Antigonish, NS, Canada; 2 School of Kinesiology, 8166 University of British Columbia, Vancouver, BC, Canada

**Keywords:** joint action, non-complementary task, response-discrimination

## Abstract

When paired participants are each assigned a complementary half of the Simon task, a joint Simon effect (JSE) has been observed. Co-representation, a cognitive representation of not only one’s own task but also that of the co-actor, has been one of several proposed mechanisms in the JSE. Using the response-discrimination hypothesis as a framework, we tested whether it was sufficient to highlight alternative task keys in a two-person setting in which a non-complementary task was completed to elicit a Simon effect (SE). In our design, the participant’s role was to perform the Go/No-Go Simon task and the co-actor’s role was to initiate each trial for the participant. In one two-person setting participant group (SK group), the same task key was assigned to both the participant and the co-actor; another group (OK) was assigned spatially opposite task keys. In a third group (joint setting, TS group), the standard joint Simon task was also completed to verify that a JSE could be replicated. We hypothesized that an SE would be elicited in the OK group, since opposite task keys would uniquely promote spatial coding. We found a weak but marginally significant SE in the OK group but not in the SK group. These results suggest that, on a non-complementary task, response discrimination may contribute to the emergence of a SE in a two-person setting, while it does not have the same impact as a complementary task completed in a joint setting (TS group) that may afford more robust response representations that reveal the enhanced so-called JSE.

## Introduction

Investigators of joint action have shown that a co-actor’s task can influence a participant’s action planning (see Kourtis et al., 2019; Sebanz et al., 2003; Török et al., 2021). For example, during social interaction, having advanced knowledge of another person’s task helps a participant predict and simulate their actions; this is said to be supported by co-representation (Sebanz, et al., 2003). Variants of the Simon task (e.g., Simon, 1990; Simon et al., 1970) have been one of the primary methods used to test and support ideas of co-representation. In a typical Simon task, stimuli are presented randomly to the left or right of central fixation and participants are instructed to make a left or right keypress response based on a stimulus feature other than spatial location (e.g., color or shape). The Simon effect (SE) refers to a shorter reaction time (RT) when the stimulus location is spatially compatible with the keypress location (right stimulus, right response) and a longer RT when it is not (right stimulus, left response), even though stimulus location is not explicitly relevant to the task (Simon & Ruddell, 1967).

While the SE is observed in a two-choice reaction task, it has tended to be absent (Ansorge & Wühr, 2004, 2009) or markedly reduced (Callan et al., 1974) in a one keypress location of the Go/NoGo version of the Simon task. In the Go/NoGo situation, participants respond to the Go stimulus (e.g., a circle) and withhold their response when the NoGo stimulus (e.g., a square) is presented (*cf.* Hommel, 1996). Even in this condition, however, it is possible to elicit an SE when a Go/NoGo Simon task is performed alongside another person. Sebanz et al. (2003) had participants complete the Go/NoGo Simon task in a *solo* setting in which they were instructed to make a spatially defined keypress response (e.g., left) to a specified ring color (red) and to refrain from responding to the other ring color (green). On any given trial, a hand with the index finger pointing either to the left or the right would appear at the center of the display; the ring (red or green) was on the pointing finger. Next, participants performed the same task alongside a co-actor beside them (a new social context or *joint* setting). The co-actor responded to the stimulus the participant had been instructed to ignore (green ring). This task has been described as ‘complementary’ in nature, because each participant is assigned one-half of the whole task. It is important to highlight that, in this paradigm, each participant knows the co-actor’s task, and the task is no different in either the solo or joint settings. Lastly, Sebanz et al. (2003) had participants complete the standard two-choice Simon task at the end of the testing session. As anticipated, the SE has been observed in the typical Simon task (see De Jong et al., 1994; Kornblum et al., 1990; Lu & Proctor, 1995; Prinz, 1990), but not in the Go/NoGo Simon task performed in the solo setting. However, the SE re-emerged in the joint setting (termed a joint Simon effect or JSE), in which Sebanz et al. (2003) argued that participants then generated representations of their co-actor’s task.

There have been challenges to this co-representation account, with alternative explanations for the JSE put forth. For example, Guagnano et al. (2010) offered evidence that spatial boundaries can influence the manifestation of the JSE. These authors carried out two separate experiments, the first of which investigated whether the JSE could be elicited when two participants performed *independent* tasks. In the typical joint Simon task, only one stimulus is presented on any given trial. By presenting both stimuli at the same time (i.e., simultaneously), the investigators removed “any aspect of turn-taking from the task” (Guagnano et al., 2010, p. 351), meaning that the task would no longer be collaborative. In 80% of the trials, both stimuli would appear simultaneously (double stimulus trial), while, in the remaining 20% of trials, only one stimulus appeared (single stimulus trial). A significant, yet small, JSE was still detected, even though the participants performed their tasks independently. To establish whether the observed effect was simply due to the proximity in which the participants completed the task, a second experiment was conducted in which participants sat either beyond arm’s reach (extra-personal space, Experiment 2a) or within arm’s reach (peri-personal space, Experiment 2b). The JSE only manifested when the co-actor was within one’s peri-personal space (cf. Welsh et al., 2013). Guagnano et al. (2010) argued that the co-representation account could not explain why the JSE re-emerged. Rather, they suggested that the co-actor served as a spatial reference point that allowed the two response keys to be spatially coded as left and right when participants were close but not when they sat beyond arm’s reach. Although this spatial coding account is feasible, it is important to note that participants were also performing two *independent* tasks rather than one *complementary* task, and the spatial coding account did not explain how social factors (e.g., group membership) (Iani et al., 2011), differences in race (Croker et al., 2015), and empathy and friendship (Ford & Aberdein, 2015) can modulate the JSE (but see Dolk et al., 2011; Dolk et al., 2013; Sellaro et al., 2015 for a referential coding account that can address these limitations).

How are spatial codes selected to represent responses in a task? What task requirements promote a particular spatial response representation? According to the response-discrimination hypothesis, if response keys can be spatially discriminated in a two-choice RT task, then they will be represented accordingly (Ansorge & Wühr, 2004, 2009). For example, the standard Simon task affords two response keys (left, right) that can be spatially coded, making their spatial locations relevant and valuable in representing them. In contrast, in a simple RT task, as in the Go/NoGo Simon task, spatial discrimination would be of no use. Ansorge and Wühr (2004) contended that, under such circumstances, if anything were to be represented and stored in working memory, it would be to *press* or *not press* the response. Ansorge and Wühr (Experiment 5, 2004) presented a clever demonstration of how two alternative keypress tasks can be spatially discriminated even though only one is used to achieve the goal. In the testing session, participants completed the Go/NoGo Simon task but also had to self-initiate every trial. Half the participants were assigned the *same-side* start key (SK) condition, while the other half were assigned a left key to initiate the trial and a right key to respond to the target, the *opposite-side* start key (OK) condition. An SE emerged only in the *opposite-side* start key condition when the alternative task keys could be spatially coded and represented in working memory.

In the present study, we did not intend to pit the response-discrimination account against the task or action co-representation account; rather, we sought to broaden our understanding of the conditions under which an SE^1^ can be elicited in a two-person setting with a non-complementary task. The response-discrimination hypothesis offers a framework to test whether the highlighting, or discrimination, of a spatially distinct alternative task key is enough to elicit an SE. Firstly, as the role of the co-actor in both the OK and SK groups is not to respond to a particular stimulus-action relationship, there is no *task* to co-represent. While the co-actor is making a keypress, they are not doing so in response to a particular stimulus alternative; there is no *action* to co-represent. Secondly, as only the participant is responding to the task stimulus and the co-actor does not participate during the *trial* (defined as the onset of the stimulus to the response).

We modified the task designed by Ansorge and Wühr (see Experiment 5, 2004) to accommodate the participant and the co-actor in a non-complementary task, defined as one in which we eliminated a sense of turn-taking. While, in the typical joint Simon task, the participant was assigned half the task and the co-actor the complementary half (see Sebanz et al., 2003), we assigned the co-actor and the participant non-complementary tasks in a two-person setting. The co-actor was always responsible for initiating each trial for the participant by either pressing the *same* key (SK group) as the participant or by pressing the task key that was spatially *opposite* to it (OK group). Based on the response-discrimination hypothesis, we predicted an SE in the two-person setting in the OK group. This was based on the context of the functionally opposite task keys which we expected to promote spatial discrimination. Conversely, we hypothesized an absence of the SE in the two-person setting in the SK group. This was due to the fact that the co-actor and the participant shared the same task key, and the left-right spatial codes were assumed to be less relevant. We also had the participants complete the standard joint Simon task to ensure that the JSE, as reported by Sebanz et al. (2003), could be replicated (TS group); we anticipated a JSE under these circumstances. Irrespective of group, we did not expect to observe an SE in the solo setting.

## Method

### Participants

To calculate a sample size estimation, we followed the approach outlined in a joint action study by McEllin et al. (2018) since our study also used a novel paradigm (non-complementary task). An a priori power analysis was conducted using G*Power version 3.1.9.6 software (Faul et al., 2007). We used a significance criterion of α = .05 and power = .80 and found that the minimum sample size needed with effect size was *N* = 42. This means that the minimum number of participants required per group would be 14 (3 groups). Thus, we recruited 45 students from the University of British Columbia (UBC) and St. Francis Xavier University (StFX) communities as participants, which is adequate to test the study hypothesis. Fifteen participants were assigned to the *task sharing* (TS) group (M_age_ = 22.7 years, SD = 3.5; 11 females, 4 males), fifteen were assigned to the *opposite-side* start key (OK) group (M_age_ = 23.7 years, SD = 4.6; 8 females), and fifteen were assigned to the *same-side* start key (SK) group (M_age_ = 22.9 years, SD = 3.5; 7 females, 8 males). All participants reported that they had normal or corrected-to-normal visual acuity. We obtained written informed consent from all participants. The procedures of the study were approved by the ethics committee of both UBC and StFX.

### Apparatus and Stimuli

**Figure 1.**
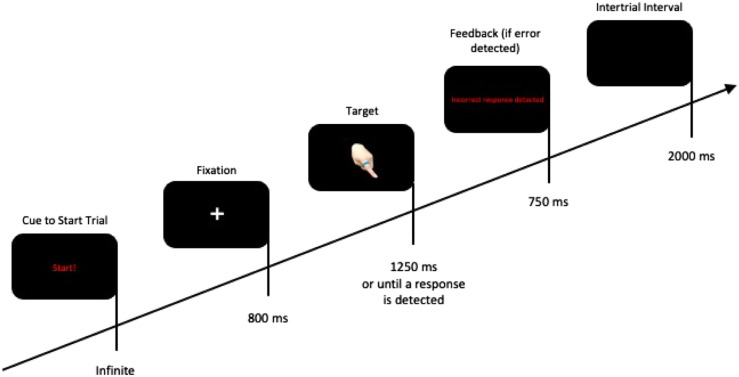
Sequence of Events Within a Trial. Note: The first event appeared in the two-person setting for the OK and the SK groups only.

Two chairs were placed in front of a table side-by-side approximately 45 cm apart and 80 cm from the computer monitor. On the table were two alternative response/task keys positioned approximately 50 cm in front of a 19″ LCD monitor. All stimuli were presented on a black background. Each trial began with a small grey cross (.5 cm × .5 cm) presented for 1500 ms at the centre of the monitor. Then, a white fixation cross (1 cm × 1 cm) was displayed for 800 ms. Like the stimuli used by Sebanz et al. (2003), digital photographs of a hand (9.5 cm × 12 cm) with a colored ring (blue or yellow; 2.2 cm × 1.6 cm) on the index finger (8 cm × 2 cm) pointing to the left or right. The colored ring always appeared at the center of the monitor. The stimulus remained on the monitor for a maximum of 1250 ms, or until the participant responded. Immediately after a response was detected, the target disappeared. An incorrect response generated a feedback message that was presented for 750 ms. The intertrial interval was set at 2000 ms. This sequence of events was the same except for the OK and SK groups in the two-person setting; trials started with a cue to start with an infinite time interval (see Figure 1). A computer running E-Prime V3.0 software (Psychology Software Tools, Pittsburgh, PA) was used to program the experiment and collect data.

## Procedure

**Figure 2.**
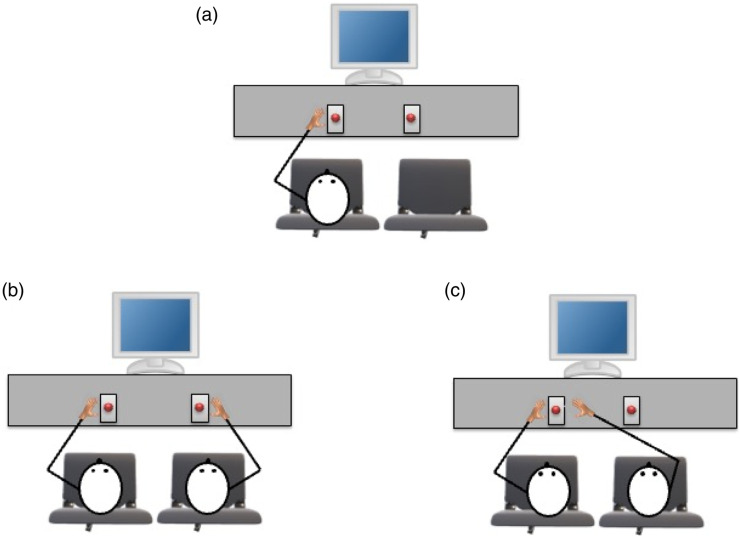
Schematic of the Experimental Setup in the: (A) Solo setting, (B) Two-Person setting for the *Opposite-Side* Start Key Group and the Joint setting for the *Task Sharing* Group, and (C) Two-Person setting for the *Same-Side* Key Group.

Participants were asked to sit in one of two chairs (left or right). Their chair determined which hand they would use to respond (e.g., left seat, left index finger). All participants first completed a Go/NoGo Simon task in the solo setting (see Figure 2(A)). The instructions were given, and a stimulus-response mapping was assigned to them. They were told to press their assigned response key as quickly and as accurately as possible when the stimulus assigned to them (e.g., blue ring) appeared on the monitor; they were asked to ignore the other stimulus (e.g., yellow ring). Participants were informed that the direction that the finger of the hand pointed was irrelevant to the task.

After completing the Go/NoGo Simon task in the solo setting, participants were introduced to the co-actor (a confederate) who sat in the seat beside them. In the TS group, the co-actor was instructed to place their index finger on the opposite response key to the participant (see Figure 2(B)). The experimenter reminded the participant and the co-actor to perform the same task as they had just completed in the solo setting. The participant and the co-actor were always assigned different stimulus-response mappings.

In the OK and SK groups, the co-actor also sat next to the participant in the other chair. The experimenter told the participant they were completing the same task as in the solo setting. They introduced the co-actor and explained that their role was to initiate each trial for the participant by pressing their assigned task key. In contrast to the TS group, the participant and the co-actor were assigned unique roles in the task; the task was no longer complementary in nature. At the beginning of each trial the word “*Start!”* would appear at the center of the monitor (see the note in Figure 1) acting as a cue for the co-actor to press their task key to initiate the trial for the participant. In the OK group, the co-actor pressed the opposite task key to the participant (see Figure 2(B)). In the SK group, the co-actor pressed the same response/task key as the participant (see Figure 2(C)).

Participants completed 10 practice trials followed by 20 blocks of 20 test trials in both the solo setting and the two-person/joint setting. The blue and yellow rings were equally distributed across the index fingers pointing to the left and the right within any given block. Participants completed 400 test trials in the solo setting and 400 test trials in the two-person/joint setting, for a total of 800 trials. To avoid stimulus-response re-mapping, participants always responded to the same stimulus using the same response key in any given testing session. The Go/NoGo Simon task was always completed in the solo setting first to avoid potential carryover effects (see Ansorge & Wühr, 2004, 2009; Hommel, 1996). Testing was completed in a single session lasting less than 2 hours.

### Data Analysis

Responses were sorted according to stimulus color (yellow, blue), and the direction that the index finger was pointing (left, right). Trials were eliminated from the data set if an incorrect response was made or if no response was detected. If the RT on any given trial was two standard deviations (*SD*s) above or below the participant’s mean for that trial type and setting, then they were also eliminated from the analysis. Consequently, .021% (246 trials), .019% (225 trials), and .019% (226 trials) were removed from the TS, OK, and SK groups, respectively. None of the participants exceeded a 1.0% (8 trials) error rate across all trials.

We analyzed the data in the solo (individual Go/NoGo Simon task) and two-person/joint (two-person/joint task) settings separately. The individual Go/NoGo Simon task is often run to show that the SE would not emerge (e.g., Sebanz et al., 2003; Tsai et al., 2006). Thus, we also expected that there would be no SE in this solo situation. The individual Go/NoGo Simon task data from the three participant groups were analyzed in a 3 Group (TS, OK, SK) × 2 Compatibility (compatible, incompatible) ANOVA with repeated measures on the latter factor.

To determine if there was a compatibility effect in the two-person/joint setting in the three different participant groups, we submitted the mean RTs from these settings to a 3 Group (TS, OK, SK) × 2 Compatibility (compatible, incompatible) ANOVA with repeated measures on the latter factor. We expected that an SE would be observed in the “joint” situation (i.e., TS group) - the so-called “joint Simon effect” (e.g., Sebanz et al., 2003). Based on predictions of the response-discrimination account, we expected an SE in the “two-person” situation for the OK group, but not in the “two-person” situation for the SK group. When necessary, post hoc pairwise comparisons with Holm adjustments were conducted. Partial eta-squared (η_p_^2^) statistics are reported for effect sizes. Statistical significance was set at an α of .05 for all analyses.

## Results

The mean error rate in the solo setting was negligible: .0002% (1 trial), .0002% (1 trial), and .0002% (1 trial) in the TS, OK, and SK groups, respectively. They were also minimal in the joint setting in the TS group (.0070%, 42 trials) and the two-person setting in the OK (.0010%, 6 trials) and SK (.0007%, 4 trials) groups.

We verified the expected absence of an SE effect in the solo setting. The analysis indicated no main effects for Compatibility, (F(1, 42) = .051, *p* = .822, η_p_^2^ = .001), or Group, (F(2, 42) = .514, *p* = .602, η_p_^2^ = .024), and no significant interaction effect (F(2, 42) = 1.85, *p* = .170, η_p_^2^ = .081). This indicated that when participants performed the Go/No-Go Simon task, the SE did not emerge.

There was a main effect for Compatibility, (F(1, 42) = 9.729, *p* = .003, η_p_^2^ = .188) but not for Group, (F(2, 42) = .442, *p* = .646, η_p_^2^ = .021). There was a significant interaction between Compatibility and Group, (F(2, 42) = 4.094, *p* = .024, η_p_^2^ = .163). Given that we expected that RTs for compatible trials would be shorter than incompatible trials (i.e., the compatibility effect), we performed one-tailed post-hoc comparisons (Holm-adjusted) to examine compatibility effects within each group. Specifically, we hypothesized that RTs for the compatible trials would be significantly shorter than the incompatible trials in the OK and TS groups. In contrast, we anticipated no difference in RTs for the compatible and incompatible trials in the SK group. They indicated that the TS group showed a significantly shorter RT on compatible compared to incompatible trials (*p* = .006). The OK group showed a similar pattern, albeit smaller and marginally significant (*p* = .036). The SK group did not show a significant difference (*p* = .968).

## Discussion

According to the response-discrimination hypothesis, an SE can be observed in a Go/NoGo Simon task if alternative response keys are spatially discriminated from one another (see Ansorge & Wühr, 2004). We explored whether this prediction could also apply to a Go/NoGo Simon task when completed in a two-person setting. Ansorge and Wühr (2004) noted an SE in a Go/NoGo Simon task when the key to initiate each trial and the response key to respond to the target stimulus could be discriminated along a left-right dimension (spatially opposite each other). When the same response key was used to complete both tasks, the SE was noticeably absent. They made the case that an SE can arise when the task context affords the spatial coding of the response keys. Based on these findings, we aimed in the present study to test the predictions made by the response-discrimination hypothesis in a non-complementary task completed in a two-person setting.

The two-person setting provides a social context (i.e., completing the task alongside a co-actor) for participants in both the OK and SK groups much like the joint setting in the TS group. What set the OK and SK groups apart from the TS group was the role of the co-actor. Rather than assigning them a complementary Go/NoGo Simon task, the co-actor initiated each trial by pressing a task key opposite to the participant (OK group) or pressed the same task key as the participant (SK group). The nature of the preceding tasks (*non-complementary* rather than c*omplementary*) and different task goals (co-actor initiating the trial for the other rather than responding to the complementary stimulus) may not be supported by the same mechanism (co-representation) used in the joint setting for the TS group (Knoblich & Sebanz, 2006). Co-representation facilitated mutual action prediction and adjustments to movement behaviour (Sebanz et al., 2006) and supported shared responsibility for achieving task goals. For this reason, the weak SE observed in the two-person setting in the OK group was not necessarily surprising.

Alternatively, the response-discrimination hypothesis would predict that assigning opposite task keys (OK group) should promote spatial coding along the left-right dimension but not when the same task key (SK group) was assigned in the two-person setting. These predictions received some support in our study, as reflected by the small difference between compatible and incompatible trials in the two-person setting in the OK group. The net compatibility effect in the two-person setting in the OK group was 5.3 ms; the SK group showed no net compatibility effect (−0.1 ms) in the two-person setting. As predicted, a JSE was observed in the joint setting in the TS group providing continued support for co-representation in a *complementary* task. The net JSE was 14.1 ms, which is comparable to the 11.0 ms reported by Sebanz et al. (2003).

Perhaps our two-person setting offered less than favorable conditions for response discrimination based on spatial coding. Since the participants in Ansorge and Wühr’s study (Experiment 5, 2004) completed the task in isolation (i.e., solo setting), they were responsible for both the initiation of the trial *and* the decision to respond to the target or not. This dual role may have encouraged more focused attention to the whole task to ensure successful completion. And while these two roles did not overlap (i.e., two duties were not performed simultaneously as in a dual-task paradigm), they required a degree of orderliness (i.e., keeping in mind what needs to be performed next: an initiation or a response). Consequently, the discrimination of horizontal spatial codes (OK condition) became increasingly salient. In contrast, the non-complementary nature of our task in the two-person setting weakly supported spatial discrimination in the OK group. A potential reason for this may be that when the co-actor (a confederate) and participant were given distinct roles in a ‘shared’ spatial coding of task keys was attenuated because there was no longer the level of response conflict to delay response decision as in the TS group where the Simon task was shared.

A point of contention for some might be our claim that the participant and the co-actor were performing non-complementary tasks. After all, they were taking turns and participants could not achieve *their* task goal without the co-actor, because the trial would not be initiated without a co-actor. While a valid criticism, the co-actor’s role did not directly impact the participant’s success at responding to the assigned stimulus while ignoring the other (Go/NoGo task). Initiating the trial quickly or slowly would not adversely affect the participant’s performance. In the TS group, the participant and the co-actor’s roles complemented each other on the Simon task itself. For example, when a blue ring appeared on the finger, the participant responded, and the co-actor refrained from responding, and vice versa. The participant and the co-actor accepted the turn-taking involved in the *shared* Simon task; each was responsible for one-half of the task to complete the whole task.

The joint Simon task (Sebanz et al., 2003) is complementary in nature. Participants are responding to stimuli in a complementary manner; this offers an interactive partnership. In contrast, in our study (two-person setting in the OK and SK groups) the participant never engaged in a joint Simon task (no “partner” complementing the task) but rather performed the Go/NoGo Simon task alongside the co-actor who simply initiated each trial for them. In the latter scenario, the co-actor may have been perceived as having a peripheral role in the overall task. This may explain the weak effect we observed. Consequently, the participant treated the task differently and the inclination to spatially discriminate the task keys may not have carried the same weight. The key difference between the joint setting in TS group and the novel two-person setting in the OK and SK groups was that the former requires the co-actor and participant to share of the task while the latter is contingent on the co-actor simply commencing the trial. A shared representation of the Simon task may not have been formed in the two person-setting in the OK and SK groups because the Simon task is never shared with the co-actor. Future research should continue to explore how the nature of the task and the role of the co-actors shape joint action performance.

### Limitations and Directions for Future Research

The significance of the co-actor in initiating each trial, and the influence of this action in potentially prompting spatial response discrimination by the actor participant was not directly tested in the present study. That is, we do not know whether the (weak) effect we observed in the two-person setting in the OK group was dependent on the co-actor. Despite the clear absence of an effect in the two-person setting in the SK condition, our study did not directly address the significance of a co-actor leading the task in general. There are various scenarios in which this matter could be further explored.

Traditionally, the joint Simon task is preceded by a solo Go/NoGo Simon task (see Mendl et al., 2018; Sebanz et al., 2003; Wen & Hsieh, 2015). The Simon task is distributed between two participants which results in each person being assigned one response key and one stimulus color. Typically, the solo Go/NoGo Simon task is completed prior to the joint Simon task and no SE is observed when a co-actor is absent. When the co-actor is brought in and responds alongside the participant to complete their half of the task, the joint Simon effect arises. This finding provided the evidence needed to support co-representation (Sebanz et al., 2003; but also see Dolk et al., 2013 for a referential coding account). While participants did complete the Go/NoGo Simon task in a solo setting, we did not design a situation where there was no co-actor. For example, we could consider a task where the trials are initiated by simply highlighting the task key. For example, participants would be told that the trials would be initiated by the computer which would be indicated by a flash of the task key (either the same task key or the opposite task key). Such a situation would allow us to determine the significance of the co-actor’s presence.

Changing the role of the co-actor is another way that their significance could be explored in the task. The co-actor was responsible for initiating the trial for the participant in the OK and SK conditions. Rather than initiating the trial, what if the co-actor was responsible for terminating the trial? By having them terminate the trial, the temporal dynamics of the task would be altered. If the spatial discrimination of the alternative response key is dependent on the co-actor leading the task, then there should be no effect when the co-actor terminates the trial. In the case that there is no effect, as observed in the SK condition, then we would conclude that there is temporal significance in the co-actor initiating the trial.

Another possibility to explore further would be to pair two participants and have them alternate their roles. The aim would be to examine how an actor’s role (as one who initiates the trial, or as the one who performs the RT task) might influence their joint action performance. For example, in the first block of trials, Actor 1’s role would be to start the trial sequence for Actor 2, who would perform the Go/NoGo Simon task. In a subsequent block of trials, Actors 1 and 2 would switch roles, meaning Actior 1 would now perform the Go/NoGo Simon task, and Actor 2 would start the trial. In a final block of trials, Actors 1 and 2 would complete the traditional joint Simon task (complementary shared task). This within-subject design would allow for a comparison between the size of the JSE of the actors when performing in the non-complementary task condition, versus in the shared task condition.

## Conclusion

Our results from this study on iterations of the SE suggest that, in a non-complementary task, the introduction of a co-actor who pressed the opposite task key to the participant (OK group) elicited a weak but marginally significant compatibility effect. By modifying Ansorge and Wühr’s (2004, Experiment 5) task to be completed in a two-person setting, we provided an opportunity for participants to use horizontal spatial codes to discriminate between the two task keys (OK group) and found a modest compatibility effect. This finding suggests that, while response discrimination may contribute to the emergence of a compatibility effect (e.g., Shi & Wang, 2022) in a two-person setting (OK group), it does not have the same impact as a complementary task carried out in a joint setting (TS group). In the joint setting (TS group), the turn-taking, complementary parts of the Simon task are distributed. Shared representations presumably give rise to response conflict (Sebanz et al., 2003). Thus, it is reasonable for participants to represent the alternative response keys in terms of spatial codes, resulting in a more robust response representation and therefore a stronger compatibility effect. Essentially, the complementary two-person task sharing context enhances the compatibility effect. It is also important to highlight the clear absence of a compatibility effect in the two-person setting when the same task key (SK group) was pressed as it illustrates the irrelevance of response location in the discrimination of task keys. In the end, the take-home message of this study is that if the alternative response is considered - whether it be via shared representation, spatial coding, or response discrimination - it can potentially contribute to the emergence of an SE in a Go/NoGo task.

## Note

1. The anticipated effect will be described as an SE rather than a JSE because there is no task sharing in the joint setting. As such, the use of the term JSE would be misleading. The term JSE will be reserved to describe the effect identified by Sebanz et al. (2003).
